# Supplementary material for: Factors Impacting σ- and π-Hole Regions as Revealed by the Electrostatic Potential and Its Source Function Reconstruction: The Case of 4,4′-Bipyridine Derivatives
Source: Molecules. 2020 Sep 25;25(19):4409. doi: 10.3390/molecules25194409 (PMC7582854; doi:10.3390/molecules25194409)
Supplement: Supplementary file 1 [file molecules-25-04409-s001.pdf]

Supplementary information

Factors impacting σ- and π-hole regions as revealed by the electrostatic potential and its source function reconstruction: the case of 4,4′-bipyridine derivatives

Carlo Gatti [1,2,*], Alessandro Dessì [3], Roberto Dallocchio [3], Victor Mamane [4,*], Sergio Cossu [5], Robin Weiss [4], Patrick Pale [4], Emmanuel Aubert [6], and Paola Peluso[3,*]

1 CNR-SCITEC, Istituto di Scienze e Tecnologie Chimiche “Giulio Natta”, sezione di via Golgi, via C. Golgi 19, 20133 Milano, Italy
2 Istituto Lombardo, Accademia di Scienze e Lettere, via Brera 28, 20100 Milano, Italy
3 Institute of Biomolecular Chemistry ICB, CNR, Secondary branch of Sassari, Traversa La Crucca 3, Regione Baldinca, Li Punti, 07100 Sassari, Italy; alessandro.dessi@cnr.it (A.D.); roberto.dallocchio@cnr.it (R.D.)
4 Strasbourg Institute of Chemistry, UMR CNRS 7177, Team LASYROC, 1 rue Blaise Pascal, University of Strasbourg, 67008 Strasbourg, France; ppale@unistra.fr (P.P.); robin.weiss@unistra.fr (R.W.)
5 Department of Molecular Science and Nanosystems DSMN, Venice Ca’ Foscari University, Via Torino 155, 30172 Mestre Venezia, Italy; cossu@unive.it (S.C.)
6 Crystallography, Magnetic Resonance and Modelling (CRM2), UMR CNRS 7036, University of Lorraine, Bd des Aiguillettes, 54506 Vandoeuvre-les-Nancy, France; emmanuel.aubert@univ-lorraine.fr (E.A.)

Table of contents

**Table S1**	Energies and structural properties of low energy optimized (B3LYP/6-311G*) conformers of compounds **1-6**	Page 2
**Fig S1**	V_s representations on electron density isosurfaces (0.002 au) for conformers of compounds **2**, **3**, and **5**	Page 3
**Fig S2**	Se···N ChB in crystal packing of conformer **5**-B2 (CCDC no. 1963860)	Page 3
**Table S2**	$V_{s,max}$ calculated for conformer **6**-B1 with different method/basis set	Page 4
**Fig S3**	Linear correlation between σ- and π-holes calculated with different methods/basis set	Page 4
**Table S3**	Energies and structural properties of low energy optimized B3LYP/6-311G* conformers of compound **7**	Page 5
**Table S4**	$V_{s,max}$ on halogen (Cl, Br) and π-holes calculated for conformers **7**-A1, **7**-A2, **7**-B1, and **7**-B2	Page 5
**Table S5**	$V_{s,max}$ and their Source Function (SF) atomic group contributions on external and internal π-holes	Page 6
**Table S6**	$V_{s,max}$ variations ($\Delta V_{s,max}$) of $C_{pyridyl}$-Ch and C_R-Ch (Ch = S, Se) σ-holes and corresponding Source Function (SF) contributions changes (ΔSF) upon substitution of Ch or R atom/group or of both of them for a given conformer of systems **1-6**.	Page 7
**Table S7**	$\Delta V_{s,max}$ and their ΔSF atomic group contributions on $C_{pyridyl}$-Ch and on C_R-Ch σ-holes calculated upon change of conformation for systems **1-6**	Page 8

Table S1 Energies and structural properties of low energy optimized (B3LYP/6-311G*) conformers of compounds **1-6** (Spartan' 10).

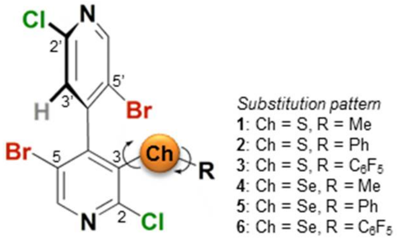

Bipyridine	Conformer	Energy [au]	Boltzmann distribution%	Dihedral angle$_1$ (°) (C3'-C4'-C4-C3)	Dihedral angle$_2$ (°) (C4-C3-Ch-C$_R$)
**1**	A1	-6999.32901	44.4	-92.5	108.8
	A2	-6999.32922	55.6	-89.7	-113.8
**2**	A1	-7191.10208	37.6	-95.5	80.6
	B1	-7191.10173	26.0	-91.5	120.6
	A2	-7191.10023	5.4	-78.1	-76.0
	B2	-7191.10189	31.0	-83.7	-127.4
**3**	A1	-7687.38855	4.5	-98.1	73.1
	B1	-7687.39137	88.1	-92.2	123.7
	A2	-7687.38796	2.4	-77.0	-71.9
	B2	-7687.38867	5.1	-79.9	-134.2
**4**	A1	-9002.65975	41.1	-91.8	112.1
	A2	-9002.66009	58.9	-90.4	-120.4
**5**	A1	-9194.43158	90.9	-93.5	84.2
	A2	-9194.42941	9.1	-81.5	-83.8
**6**	A1	-9690.71970	3.4	-99.1	73.3
	B1	-9690.72279	90.0	-95.7	122.0
	A2	-9690.71868	1.2	-77.6	-72.9
	B2	-9690.72014	5.4	-84.4	-133.5

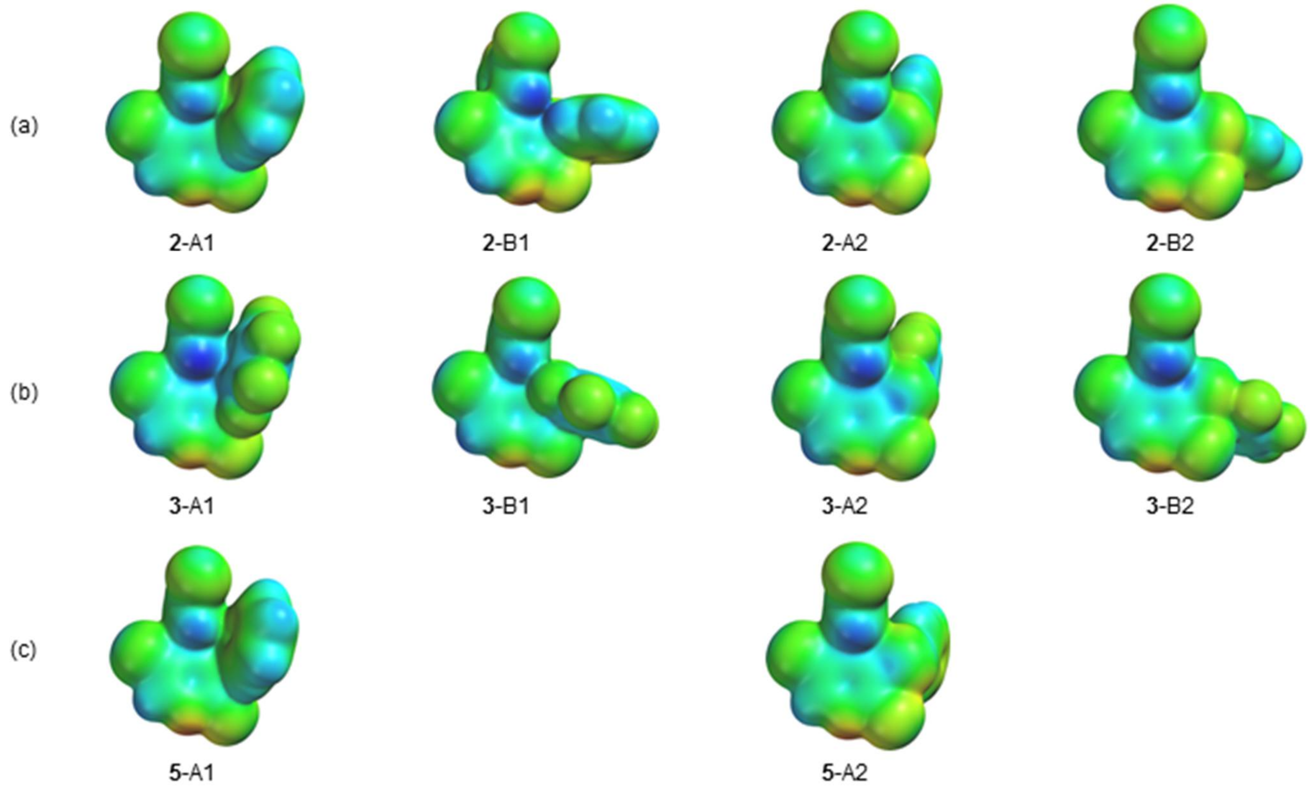

Fig. S1 V_s representations on electron density isosurfaces (0.002 au) graphically generated by using Spartan' 10 (DFT/B3LYP/6-311G*) for conformers of compounds (a) **2**, (b) **3**, and (c) **5**. For the V_s representations, colours towards red depict negative V_s, while colours towards blue depict positive V_s, and colours in between (orange, yellow, green) depict intermediate values.

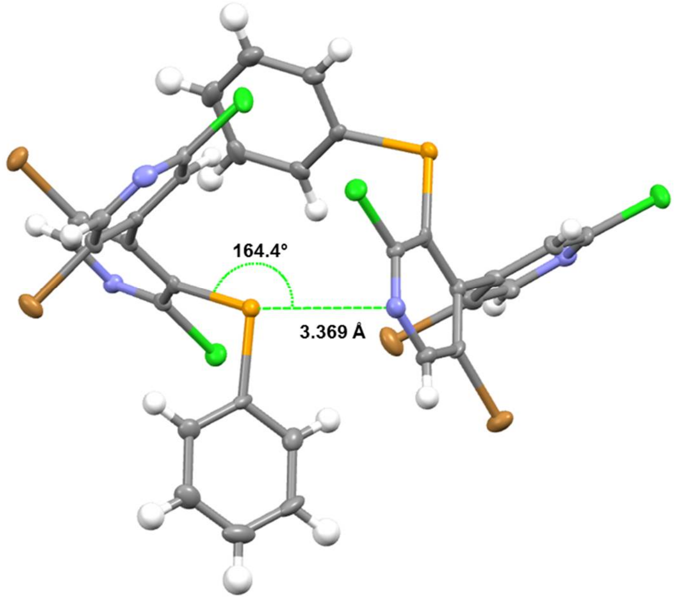

Fig. S2 Se···N ChB observed in the crystal packing of conformer **5**-B2 (CCDC no. 1963860, R. Weiss et al., *Molecules*, 2019, 24, 4484. doi: 10.3390/molecules24244484).

Table S2 $V_{s,max}$ [au] on halogens and selenium σ-holes (0.002 au molecular surface), and the pentafluorophenyl ring π-hole calculated for conformer **6**-B1, as a representative case, with different method/basis set. In parentheses the relative values normalized with respect to the values of 2-Cl' are given.

Conf.	method/basis set	2'-Cl	2-Cl	5'-Br	5-Br	Se ($C_{pyridyl}$-Se)	Se (C_{ArF}-Se)[a]	π-hole
**6**-B1	HF/3-21G*	0.0266 (1.00)	0.0330 (1.24)	0.0567 (2.13)	0.0575 (2.16)	0.0667 (2.50)	0.0296 (1.11)	0.0585 (2.20);0.0546 (2.05)
	HF/6-311G*	0.0255 (1.00)	0.0326 (1.28)	0.0516 (2.02)	0.0538 (2.11)	0.0673 (2.64)	0.0435 (1.70)	0.0504 (1.98);0.0482 (1.89)
	B3LYP/6-311G*	0.0235 (1.00)	0.0294 (1.25)	0.0496 (2.11)	0.0510 (2.17)	0.0582 (2.47)	0.0318 (1.35)	0.0455 (1.94);0.0425 (1.81)
	B3LYP/def2-QZVP	0.0251 (1.00)	0.0317 (1.26)	0.0468 (1.86)	0.0480 (1.91)	0.0564 (2.25)	0.0332 (1.32)	0.0405 (1.61);0.0367 (1.46)
	M06-2X/6-311G*	0.0249 (1.00)	0.0296 (1.19)	0.0492 (1.98)	0.0504 (2.02)	0.0565 (2.27)	0.0223 (0.89)	0.0421 (1.69);0.0387 (1.55)
	M06-2X/def2-QZVP	0.0264 (1.00)	0.0318 (1.20)	0.0467 (1.77)	0.0474 (1.79)	0.0538 (2.04)	0.0203 (0.77)	0.0356 (1.35);0.0310 (1.17)

[a] As reported in Table 1 (main text), a third σ-hole on the elongation of the C_{ArF}-Se was found for the conformer **6**-B1.

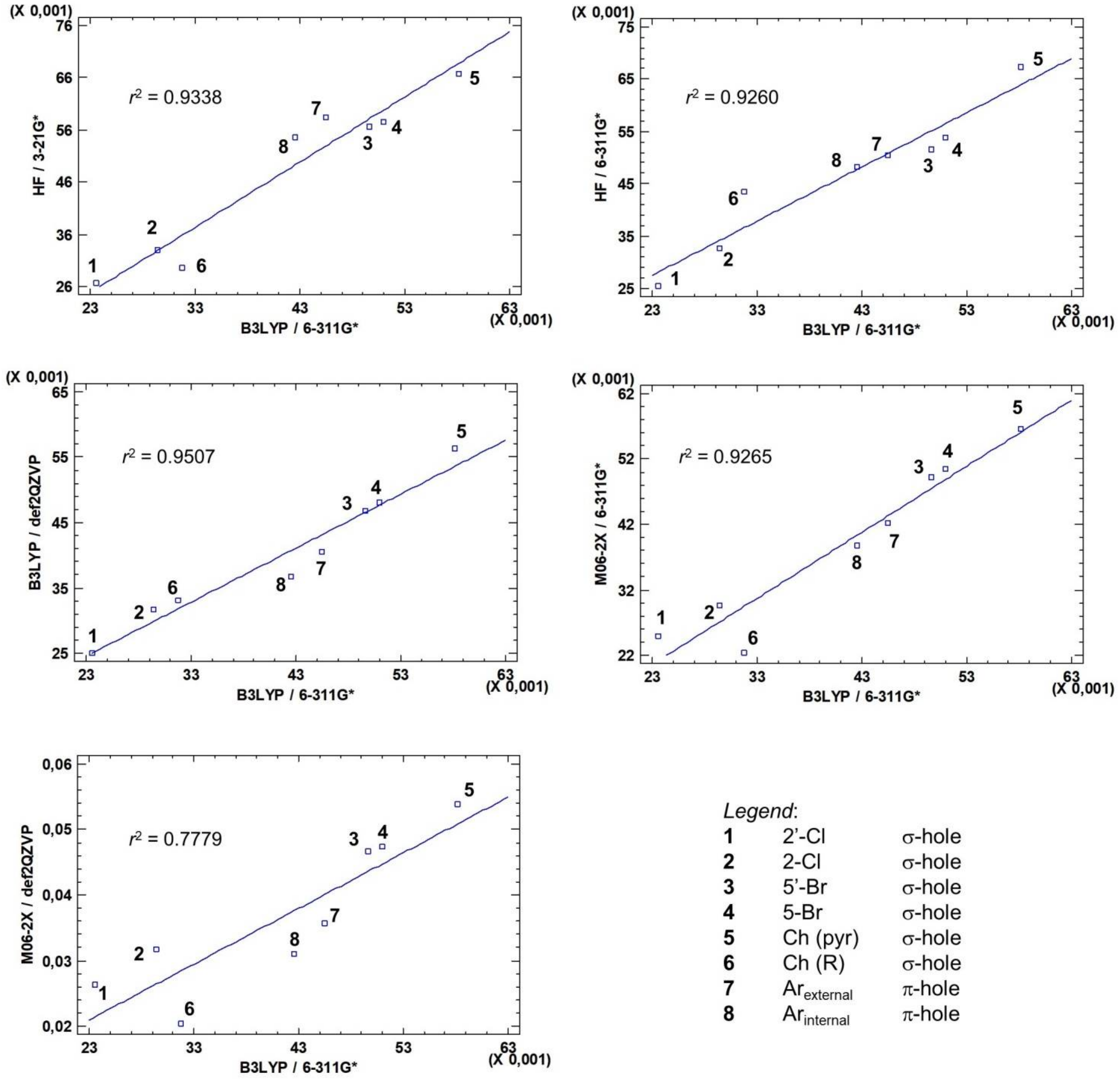

Fig. S3 Linear correlation between σ- and π-holes calculated with different methods/basis (data set reported in Table S2).

Table S3 Energies and structural properties of low energy optimized B3LYP/6-311G* conformers of compound **7** (Spartan' 10 Version 1.1.0).

Conformer	Energy [au]	Boltzmann distribution%	Dihedral angle$_1$ (°) (C3'-C4'-C4-C3)	Dihedral angle$_2$ (°) (C4-C3-CH$_2$-C$_R$)
A1	-7328.51191	11.6	-97.8	69.1
B1	-7328.51375	81.7	-98.8	117.5
A2	-7328.51124	5.7	-79.3	-71.0
B2	-7328.50954	0.9	-75.1	-126.8

Table S4 $V_{s,max}$ [au] on halogen (Cl, Br) and π-holes (0.002 au molecular surface) calculated for conformers **7**-A1, **7**-A2, **7**-B1, and **7**-B2 (B3LYP/6-311G*).

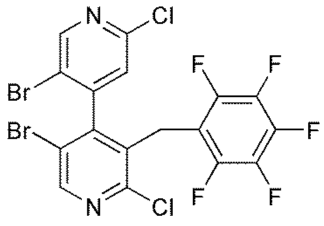

7

Conf.	2'-Cl	2-Cl	5'-Br	5-Br	π-hole (ArF)
A1	0.0256	0.0271	0.0511	0.0491	0.0418; 0.0459
B1	0.0252	0.0277	0.0509	0.0488	0.0369; 0.0416
A2	0.0259	0.0274	0.0502	0.0489	0.0409; --
B2	0.0273	0.0287	0.0479	0.0490	0.0340; 0.0398

Table S5. $V_{S,max}$ [au] and their Source Function (SF) atomic group contributions on external and internal π-holes (0.002 au molecular surface) calculated for the various conformers of systems **2**-**3** and **5**-**6**. In parentheses the SF percentage values are reported. The Err% value (see text) provides a measure of the SF $V_{S,max}$ reconstruction accuracy.

Conf.	$V_{S,max}$	SF(Ch)	SF(R)	SF(BiPy)	SF(pyr)	SF(pyr′)	SF(5′-Br)	SF(3′-H)	Err%
**π-hole, external, Ch = S**									
**2**-A1	-0.0081	0.0283 (-350.1)	-0.0288 (356.6)	-0.0066 (81.9)	-0.0113 (140.3)	0.0047 (-58.4)	-0.0007 (9.1)	0.0103 (-127.4)	11.6
**2**-B1	-0.0115	0.0336 (-291.4)	-0.0288 (250.2)	-0.0157 (136.4)	-0.0218 (189.3)	0.0061 (-52.8)	-0.0015 (12.7)	0.0082 (-71.0)	4.8
**2**-A2	-0.0090	0.0271 (-301.8)	-0.0279 (310.9)	-0.0077 (85.6)	-0.0112 (125.3)	0.0036 (-39.7)	-0.0023 (25.9)	0.0083 (-92.9)	5.3
**2**-B2	-0.0127	0.0317 (-250.9)	-0.0257 (203.5)	-0.0178 (140.8)	-0.0225 (178.5)	0.0048 (-37.7)	-0.0038 (30.3)	0.0076 (-59.9)	6.7
**3**-A1	0.0466	0.0417 (89.5)	0.0050 (10.8)	0.0006 (1.3)	-0.0047 (-10.2)	0.0053 (11.5)	-0.0003 (-0.6)	0.0098 (21.1)	-1.5
**3**-B1	0.0470	0.0422 (89.8)	0.0055 (11.7)	-0.0007 (-1.5)	-0.0067 (-14.3)	0.0060 (12.7)	-0.0010 (-2.1)	0.0111 (23.5)	-0.0
**3**-A2	0.0456	0.0413 (90.7)	0.0047 (10.4)	-0.0010 (-2.1)	-0.0046 (-10.1)	0.0036 (8.0)	-0.0036 (-8.0)	0.0087 (19.1)	1.1
**3**-B2	0.0452	0.0421 (93.0)	0.0056 (12.5)	-0.0023 (-5.1)	-0.0072 (-16.0)	0.0049 (10.8)	-0.0011 (-2.5)	0.0079 (17.4)	-0.4
**π-hole, external, Ch = Se**									
**5**-A1	-0.0070	0.0585 (-837.8)	-0.0462 (660.7)	-0.0184 (263.7)	-0.0224 (320.5)	0.0040 (-56.8)	-0.0008 (11.4)	0.0105 (-149.6	13.4
**5**-A2	-0.0081	0.0573 (-710.7)	-0.0452 (560.0)	-0.0194 (240.5)	-0.0223 (276.1)	0.0029 (-35.5)	-0.0025 (30.7)	0.0080 (-99.5)	10.2
**6**-A1	0.0454	0.0710 (156.4)	-0.0134 (-29.6)	-0.0121 (-26.7)	-0.0168 (-37.0)	0.0047 (10.2)	-0.0005 (-1.1)	0.0097 (21.4)	-0.1
**6**-B1	0.0455	0.0713 (156.8)	-0.0122 (-26.7)	-0.0140 (-30.7)	-0.0190 (-41.9)	0.0051 (11.1)	-0.0013 (-2.8)	0.0106 (23.3)	0.7
**6**-A2	0.0448	0.0711 (158.6)	-0.0125 (-27.8)	-0.0136 (-30.3)	-0.0166 (-37.1)	0.0030 (6.8)	-0.0040 (-8.9)	0.0083 (18.5)	-0.5
**6**-B2	0.0438	0.0724 (165.2)	-0.0145 (-33.0)	-0.0148 (-33.8)	-0.0188 (-43.0)	0.0040 (9.2)	-0.0014 (-3.1)	0.0076 (17.3)	1.6
**π-hole, internal, Ch = S**									
**2**-A1	-	-	-	-	-	-	-	-	-
**2**-B1	-0.0087	0.0294 (-339.3)	-0.0303 (349.1)	-0.0075 (86.6)	-0.0113 (130.2)	0.0038(-43.6)	-0.0007 (7.7)	0.0098 (-113.1	3.5
**2**-A2	-0.0138	0.0298 (-216.1)	-0.0309 (224.3)	-0.0122 (88.8)	-0.0105 (76.6)	-0.0017 (12.3)	-0.0153 (110.9)	0.0107 (-77.6)	3.0
**2**-B2	-0.0093	0.0276 (-296.8)	-0.0273 (293.0)	-0.0090 (96.7)	-0.0115 (123.9)	0.0025 (-27.2)	-0.0044 (47.7)	0.0081 (-87.3)	7.1
**3**-A1	0.0538	0.0462 (85.9)	-0.0006 (-1.1)	0.0097 (18.0)	-0.0046 (-8.5)	0.0143 (26.6)	0.0016 (3.0)	0.0193 (36.0)	-2.8
**3**-B1	0.0444	0.0467 (105.0)	0.0065 (14.7)	-0.0082 (-18.4)	-0.0169 (-38.1)	0.0088 (19.8)	-0.0015 (-3.3)	0.0104 (23.5)	-1.3
**3**-A2	-	-	-	-	-	-	-	-	-
**3**-B2	0.0404	0.0454 (112.3)	0.0073 (18.0)	-0.0116 (-28.8)	-0.0184 (-45.6)	0.0068 (16.8)	-0.0008 (-1.9)	0.0075 (18.6)	-1.5
**π-hole, internal, Ch = Se**									
**5**-A1	-	-	-	-	-	-	-	-	-
**5**-A2	-0.0160	0.0571 (-356.5)	-0.0456 (284.5)	-0.0271 (169.1)	-0.0235 (146.8)	-0.0036 (22.2)	-0.0173 (108.3)	0.0100 (-62.6)	2.9
**6**-A1	0.0527	0.0765 (145.2)	-0.0185 (-35.2)	-0.0045 (-8.6)	-0.0187 (-35.4)	0.0141 (26.8)	0.0012 (2.3)	0.0190 (36.1)	-1.5
**6**-B1	0.0425	0.0778 (183.0)	-0.0122 (-28.6)	-0.0235 (-55.2)	-0.0315 (-74.1)	0.0080 (18.9)	-0.0017 (-4.0)	0.0103 (24.3)	0.8
**6**-A2	-	-	-	-	-	-	-	-	-
**6**-B2	0.0390	0.0756 (193.7)	-0.0128 (-32.9)	-0.0247 (-63.2)	-0.0305 (-78.2)	0.0059 (15.0)	-0.0008 (-2.1)	0.0073 (18.8)	2.3

Table S6 $V_{s,max}$ variations ($\Delta V_{S,max}$) of $C_{pyridyl}$-Ch and C_R-Ch (Ch = S, Se) σ-holes and corresponding Source Function (SF) contributions changes (ΔSF) upon substitution of Ch or R atom/group or of both of them for a given conformer of systems **1-6**. SF percentage values are reported in parentheses. Potential values and their SF reconstructions refer to the 0.002 au electron density isovalue surface. $\Delta V_{s,max}$ and their composing ΔSF contributions for a system change X→Y are evaluated as ΔZ= Z(Y)-Z(X) where Z = $V_{S,max}$ or SF. In the Table, those ΔSF and ΔSF% values that refer to Ch and R moieties undergoing a change upon the X→Y system's change are shown in **bold**). $\Delta SF_{rearrangement}$ is given by the sum of ΔSF (Bipy) and of those ΔSF(Ch) or ΔSF(R) contributions whose Ch or R moieties do not undergo a chemical substitution in the X→Y process (all terms yielding $\Delta SF_{rearrangement}$ as a sum are shown not in bold).

X→Y	$\Delta V_{S,max}$ [au]	ΔSF(Ch)	ΔSF(R)	ΔSF(BiPy)	$\Delta SF_{rearrangement}$
		**σ-hole on the elongation of $C_{pyridyl}$-Ch bond**			
		R→R'			
**1**-A1 → **2**-A1	-0.0143	0.0014 (-9.8)	**-0.0195 (136.4)**	0.0037 (-25.9)	0.0051 (-35.7)
**1**-A1 → **3**-A1	0.0063	0.0311 (493.7)	**-0.0370 (-587.3)**	0.0119 (188.9)	0.0430 (682.5)
**1**-A2 → **2**-A2	-0.0156	0.0016 (-10.3)	**-0.0212 (135.9)**	0.0033 (-21.2)	0.0049 (-31.4)
**1**-A2 → **3**-A2	0.0068	0.0315 (463.2)	**-0.0371 (-545.6)**	0.0111 (163.2)	0.0426 (626.5)
**2**-A1 → **3**-A1	0.0206	0.0297 (144.2)	**-0.0175 (-85.0)**	0.0082 (39.8)	0.0379 (184.0)
**2**-A2 → **3**-A2	0.0224	0.0299 (133.5)	**-0.0159 (-71.0)**	0.0078 (34.8)	0.0377 (168.3)
**2**-B1 → **3**-B1	0.0188	0.0296 (157.4)	**-0.0190 (-101.1)**	0.0077 (41.0)	0.0373 (198.4)
**2**-B2 → **3**-B2	0.0226	0.0304 (134.5)	**-0.0154 (-68.1)**	0.0075 (33.2)	0.0379 (167.7)
		S→Se			
**1**-A1 → **4**-A1	0.0093	**0.0552 (593.5)**	-0.0295 (-317.2)	-0.0163 (-175.3)	-0.0458 (-492.5)
**1**-A2 → **4**-A2	0.0095	**0.0553 (582.1)**	-0.0293 (-308.4)	-0.0168 (-176.8)	-0.0461 (-485.3)
**2**-A1 → **5**-A1	0.0116	**0.0582 (501.7)**	-0.0296 (-255.2)	-0.0173 (-149.1)	-0.0469 (-404.3)
**2**-A2 → **5**-A2	0.0124	**0.0576 (464.5)**	-0.0281 (-226.6)	-0.0172 (-138.7)	-0.0453 (-365.3)
**3**-A1 → **6**-A1	0.0093	**0.0571 (614.0)**	-0.0298 (-320.4)	-0.0185 (-198.9)	-0.0483 (-519.4)
**3**-A2 → **6**-A2	0.0086	**0.0568 (660.5)**	-0.0294 (-341.9)	-0.0184 (-189.5)	-0.0478 (-555.8)
**3**-B1 → **6**-B1	0.0087	**0.0567 (651.7)**	-0.0299 (-343.7)	-0.0186 (-213.8)	-0.0485 (-557.5)
**3**-B2 → **6**-B2	0.0081	**0.0582 (718.5)**	-0.0325 (-401.2)	-0.0179 (-221.0)	-0.0514 (-634.6)
		S→Se and R→R'			
**1**-A1 → **5**-A1	-0.0027	**0.0596 (-2207.4)**	**-0.0491 (1818.5)**	-0.0136 (503.7)	-0.0136 (503.7)
**1**-A1 → **6**-A1	0.0156	**0.0882 (565.4)**	**-0.0668 (-428.2)**	-0.0066 (-42.3)	-0.0066 (-42.3)
**1**-A2 → **5**-A2	0.0032	**0.0592 (1850.0)**	**-0.0493 (-1540.6)**	-0.0139 (-434.4)	-0.0139 (-434.4)
**1**-A2 → **6**-A2	0.0154	**0.0883 (573.4)**	**-0.0665 (-431.8)**	-0.0073 (-47.4)	-0.0073(-47.4)
**2**-B1 → **6**-B1	0.0275	**0.0863 (313.8)**	**-0.0489 (-177.8)**	-0.0109 (-39.6)	-0.0109 (-39.6)
**2**-B2 → **6**-B2	0.0307	**0.0886 (288.6)**	**-0.0479 (-156.0)**	-0.0104 (-33.9)	-0.0104 (-33.9)
		**σ-hole on the elongation of C_R-Ch bond**			
		R→R'			
**1**-A1 → **2**-A1	0.0075	0.0104 (138.7)	**-0.0069 (-92.0)**	0.0040 (53.3)	0.0144 (192.0)
**1**-A1 → **3**-A1	0.0252	0.0322 (127.8)	**-0.0237 (-94.0)**	0.0164 (65.1)	0.0430 (192.9)
**2**-A1 → **3**-A1	0.0177	0.0218 (123.2)	**-0.0168 (-94.9)**	0.0124 (70.1)	0.0342 (193.2)
**2**-A2 → **3**-A2	0.0160	0.0222 (138.8)	**-0.0170 (-106.3)**	0.0104 (65.0)	0.0326 (203.8)
**2**-B1 → **3**-B1	0.0103	0.0173 (168.0)	**-0.0160 (-155.3)**	0.0086 (84.3)	0.0259 (251.5)
	0.0033	-0.0075 (-227.3)	**-0.0162 (-490.9)**	0.0266 (806.1)	0.0191 (578.8)
		S→Se			
**1**-A1 → **4**-A1	0.0067	**0.0472 (704.5)**	-0.0164 (-244.8)	-0.0242 (-361.2)	-0.0406 (-606.0)
**2**-A1 → **5**-A1	0.0084	**0.0518 (616.7)**	-0.0173 (-205.9)	-0.0262 (-311.9)	-0.0435 (-517.9)
**3**-A1 → **6**-A1	0.0114	**0.0578 (507.0)**	-0.0171 (-150.0)	-0.0295 (-258.8)	-0.0466 (-408.8)
**2**-A2 → **5**-A2	0.0074	**0.0514 (694.6)**	-0.0173 (-233.8)	-0.0267 (-360.8)	-0.0440 (-594.6)
**3**-A2 → **6**-A2	0.0109	**0.0569 (522.0)**	-0.0167 (-153.2)	-0.0291 (-267.0)	-0.0458 (-420.2)
**3**-B1' → **6**-B1	0.0037	**0.0324 (875.7)**	-0.0167 (-451.4)	-0.0124 (-335.1)	-0.0291 (-786.5)
	0.0047	**0.0496 (1055.3)**	-0.0169 (-359.6)	-0.0282 (-600.0)	-0.0451 (-959.6)
**3**-B1''→ **6**-B1	0.0107	**0.0572 (534.6)**	-0.0165 (-154.2)	-0.0304 (-281.1)	-0.0469 (-438.3)
	0.0117	**0.0744 (635.9)**	-0.0167 (-142.7)	-0.0462 (-394.9)	-0.0629 (-537.6)
**3**-B2 → **6**-B2	0.0050	**0.0536 (1072.0)**	-0.0177 (-354.0)	-0.0305 (-610.0)	-0.0482 (-964.0)
		S→Se and R→R'			
1-A1 → 5-A1	0.0159	**0.0622 (391.2)**	**-0.0242 (-152.2)**	-0.0222 (-139.6)	-0.0222 (-139.6)
1-A1 → 6-A1	0.0366	**0.0900 (245.9)**	**-0.0408 (-111.5)**	-0.0131 (-35.8)	-0.0131 (-35.8)
2-B1 → 6-B1	0.0140	**0.0497 (355.0)**	**-0.0327 (-233.6)**	-0.0038 (-27.1)	-0.0038 (-27.1)
	0.0150	**0.0669 (446.0)**	**-0.0329 (-219.3)**	-0.0196 (-130.7)	-0.0196 (-130.7)

Table S7 $\Delta V_{s,max}$ [au] and their Source Function (ΔSF) atomic group contributions upon change of conformation for systems **1**-**6**. Data are shown for the $C_{pyridyl}$-Ch and the C_R-Ch (Ch = S, Se) σ-holes (calculated on the 0.002 au electron density isovalue surface). $\Delta V_{s,max}$ and the various ΔSF values for a conformational change X→Y are evaluated as ΔZ = Z(Y)-Z(X) where Z = $V_{S,max}$ or SF.

X→Y	$\Delta V_{S,max}$ [au]	ΔSF(Ch)	ΔSF(R)	ΔSF(BiPy)
		**σ-hole on the elongation of $C_{pyridyl}$-Ch bond**		
**1**-A1 → **1**-A2	-0.0005	-0.0021	0.0008	0.0006
**2**-A1 → **2**-B1	0.0022	0.0010	0.0028	-0.0018
**2**-A1 → **2**-B2	0.0001	-0.0007	0.0015	-0.0016
**2**-A1 → **2**-A2	-0.0018	-0.0019	-0.0005	0.0002
**2**-B1 → **2**-A2	-0.0040	-0.0029	-0.0033	0.0020
**2**-B1 → **2**-B2	-0.0021	-0.0017	-0.0013	0.0002
**2**-A2 → **2**-B2	0.0019	0.0012	0.0020	-0.0018
**3**-A1 → **3**-B1	0.0004	0.0009	0.0013	-0.0023
**3**-A1→ **3**-A2	0.0009	-0.0049	0.0076	-0.0023
**3**-A1 → **3**-B2	0.0021	0.0000	0.0036	-0.0023
**3**-B1 → **3**-A2	0.0005	-0.0058	0.0063	0.0000
**3**-B1→ **3**-B2	0.0017	-0.0009	0.0023	0.0000
**3**-A2→ **3**-B2	0.0012	0.0049	-0.0040	0.0000
**4**-A1→ **4**-A2	-0.0003	-0.0020	0.0014	0.0001
**5**-A1→ **5**-A2	-0.0010	-0.0025	0.0010	0.0003
**6**-A1 → **6**-B1	-0.0002	0.0005	0.0012	-0.0023
**6**-A1 → **6**-A2	-0.0007	-0.0020	0.0015	0.0001
**6**-A1→ **6**-B2	0.0009	0.0011	0.0011	-0.0017
**6**-B1→ **6**-A2	-0.0005	-0.0025	0.0003	0.0023
**6**-B1→ **6**-B2	0.0011	0.0006	-0.0003	0.0007
**6**-A2→ **6**-B2	0.0016	0.0031	-0.0006	-0.0016
		**σ-hole on the elongation of C_R-Ch bond**		
**2**-A1 → **2**-B1	-0.0099	-0.0074	0.0003	-0.0029
**2**-A1 → **2**-A2	0.0072	-0.0031	0.0011	0.0090
**2**-B1 → **2**-A2	0.0171	0.0043	0.0008	0.0119
**3**-A1 → **3**-B1	-0.0173	-0.0119	0.0011	-0.0067
	-0.0243	-0.0367	0.0009	0.0113
**3**-A1→ **3**-A2	0.0046	0.0005	-0.0056	0.0091
**3**-A1 → **3**-B2	0.0108	-0.0107	0.0023	0.0195
**3**-B1 → **3**-A2	0.0219	0.0124	-0.0067	0.0158
	0.0289	0.0372	-0.0065	-0.0022
**3**-B1→ **3**-B2	0.0281	0.0008	0.0012	0.0262
	0.0351	0.0256	0.0014	0.0082
**3**-A2→ **3**-B2	0.0062	-0.0116	0.0079	0.0104
**4**-A1→ **4**-A2	0.0203	0.0026	0.0009	0.0167
**5**-A1→ **5**-A2	0.0062	-0.0035	0.0011	0.0085
**6**-A1 → **6**-B1	-0.0250	-0.0373	0.0015	0.0104
	-0.0240	-0.0201	0.0013	-0.0054
**6**-A1 → **6**-A2	0.0050	-0.0036	0.0013	0.0074
**6**-A1→ **6**-B2	0.0044	-0.0153	0.0017	0.0185
**6**-B1→ **6**-A2	0.0300	0.0337	-0.0002	-0.0030
	0.0290	0.0165	0.0000	0.0128
**6**-B1→ **6**-B2	0.0294	0.0220	0.0002	0.0081
	0.0284	0.0048	0.0004	0.0239
**6**-A2→ **6**-B2	-0.0006	-0.0117	0.0004	0.0111
